# How Digital Stress and eHealth Literacy Relate to Missed Nursing Care and Willingness to Use AI Decision Support

**DOI:** 10.3390/healthcare14080996

**Published:** 2026-04-10

**Authors:** Emilia Clej, Adelina Mavrea, Camelia Fizedean, Alina Doina Tănase, Adrian Cosmin Ilie, Alina Tischer

**Affiliations:** 1 Doctoral School, “Victor Babes” University of Medicine and Pharmacy, 300041 Timisoara, Romania; emilia.clej@umft.ro; 2 Centre for Translational Research and Systems Medicine (CERT-MEDS), “Victor Babes” University of Medicine and Pharmacy, 300041 Timisoara, Romania; 3 Faculty of Nursing, “Victor Babes” University of Medicine and Pharmacy, 300041 Timisoara, Romania; mavrea.adelina@umft.ro (A.M.); fizedean.camelia@umft.ro (C.F.); 4 Multidisciplinary Heart Research Center, “Victor Babes” University of Medicine and Pharmacy, 300041 Timisoara, Romania; 5 Department of Professional Legislation in Dental Medicine, Faculty of Dental Medicine, “Victor Babes” University of Medicine and Pharmacy, 300041 Timisoara, Romania; 6 Research Centre in Dental Medicine Using Conventional and Alternative Technologies, Faculty of Dental Medicine, “Victor Babes” University of Medicine and Pharmacy, 300041 Timisoara, Romania; 7 Department of Functional Sciences, Discipline of Public Health, Center for Translational Research and Systems Medicine, “Victor Babes” University of Medicine and Pharmacy, 300041 Timisoara, Romania; 8 Ear-Nose-Throat Department, “Victor Babes” University of Medicine and Pharmacy, 300041 Timisoara, Romania; tischer.alina@umft.ro

**Keywords:** technostress, eHealth literacy, burnout, missed nursing care, artificial intelligence, clinical decision support

## Abstract

**Background**: Digitalization and artificial intelligence-supported clinical decision support systems (AI-DSS), defined here as tools that generate patient-specific alerts, risk estimates, prioritization prompts, documentation suggestions, or related recommendation outputs intended to support rather than replace professional nursing judgment, can improve clinical decision-making, yet they may also amplify technostress and burnout, with downstream effects on missed nursing care and implementation readiness. **Methods**: We surveyed 239 registered nurses from a tertiary-care hospital in Timișoara, Romania (January–March 2025), including critical care (*n* = 60) and general wards (*n* = 179). Measures included a 15-item technostress scale, eHEALS, Maslach Burnout Inventory–Human Services Survey (MBI-HSS), Safety Attitudes Questionnaire (SAQ) teamwork and safety climate subscales, a 10-item missed nursing care inventory, and a six-item AI-DSS acceptance scale reflecting perceived usefulness, trust, and stated willingness to use such tools if available as an attitudinal readiness outcome rather than as routine observed use. Multivariable regression, exploratory mediation models, cluster analysis, and exploratory ROC analysis were performed. **Results**: Higher technostress was associated with higher emotional exhaustion (r = 0.52) and more missed care (r = 0.41), whereas eHealth literacy correlated with higher AI-DSS acceptance (r = 0.35) and lower technostress (r = −0.34). In adjusted models, technostress (per 10 points) was associated with higher missed care (β = 0.28, *p* < 0.001) (equivalent to 0.14 points per 5-point increase) and higher odds of low AI-DSS acceptance (OR = 1.38, *p* = 0.001), while eHealth literacy was associated with lower odds of low acceptance (OR = 0.71 per 5 points, *p* < 0.001). Burnout and the safety climate statistically accounted for approximately 35% of the technostress–missed care association. Three workflow phenotypes were identified, with the high-strain/low-literacy cluster showing the most missed care (3.5 ± 1.8) and the lowest AI acceptance (19.7 ± 5.2). An exploratory in-sample ROC model for intention to leave achieved an AUC of 0.82. **Conclusions**: Higher technostress clustered with worse nurse well-being, more care omissions, and lower AI-DSS acceptance, whereas eHealth literacy appeared protective. Interventions combining digital skills support, usability-focused redesign, and a stronger safety climate may reduce missed care and support safer AI implementation.

## 1. Introduction

Digital transformation in hospitals has accelerated the introduction of electronic documentation, workflow platforms, and artificial intelligence-supported clinical decision support systems (AI-DSS). In this study, AI-DSS refers to digital tools that provide patient-specific alerts, risk estimates, prioritization prompts, documentation suggestions, or related recommendation outputs intended to support, rather than replace, professional nursing judgment. While these systems can standardize care processes and support decision-making, they also reshape cognitive workload, documentation burden, trust relationships, and implementation demands in everyday practice [1,2,3,4,5].

eHealth literacy describes the skills required to locate, understand, appraise, and apply digital health information to solve problems and make decisions [1,2,4,6,7,8,9]. In clinical teams, higher eHealth literacy may support more efficient navigation of digital workflows, greater confidence in interpreting outputs from electronic systems, and more informed engagement with AI-enabled recommendations [10,11,12,13,14]. The recent nursing literature also suggests that digital readiness is shaped not only by individual literacy but by training exposure, organizational support, and system usability, which makes eHealth literacy a potentially modifiable determinant of successful digital and AI adoption [3].

Technostress is the strain experienced when individuals struggle to cope with new technologies, including overload, complexity, interruptions, and expectations of constant connectivity. In this study, technostress refers specifically to perceived strain arising from digital work systems. We use the broader term digital strain in the Discussion as an interpretive umbrella that includes technostress together with related EHR burden and downstream exhaustion; by contrast, EHR stress denotes documentation-specific burden [7,8,9,15,16,17,18,19,20,21]. Among nurses, technostress has been linked to burnout and dissatisfaction, while recent work further suggests that technology competence can weaken some of its adverse pathways [7,22].

In this manuscript, burnout is treated as a multidimensional occupational syndrome encompassing emotional exhaustion, depersonalization, and reduced personal accomplishment, and it has been consistently associated with adverse staff outcomes and lower perceived quality and safety of care [11]. Nursing teams are especially vulnerable when staffing is insufficient, because higher patient-to-nurse ratios increase time pressure and reduce opportunities to complete essential care activities and cross-checks [11]. The safety culture, captured in part by instruments such as the Safety Attitudes Questionnaire (SAQ), reflects shared norms about teamwork and safety priorities and may buffer—or amplify—the downstream consequences of digital strain [10].

Missed nursing care (also called “care left undone”) is a well-established process indicator of care quality and is strongly associated with workload, staffing, and the unit climate [12,13,14]. At the same time, the recent literature on AI in nursing emphasizes that adoption depends on more than perceived utility alone: self-efficacy, digital literacy, trust, training, human factors, and implementation context all shape whether nurses view AI as a helpful augmentation or an additional burden [21,22,23,24]. This adjacent literature suggest that care omissions and AI readiness may be linked through the same work-system pressures rather than representing separate implementation problems.

Empirical evidence linking technostress, eHealth literacy, burnout, missed nursing care, and AI-DSS acceptance within the same nursing cohort remains limited, particularly in Central and Eastern European hospital settings. Accordingly, the present study had four aims: (1) to quantify associations among technostress, eHealth literacy, burnout, the safety climate, missed nursing care, and AI-DSS acceptance; (2) to identify independent correlates of missed care and low AI-DSS acceptance in adjusted models; (3) to explore whether burnout and the safety climate statistically accounted for part of the technostress–missed care association; and (4) to describe clinically interpretable workflow phenotypes defined by digital strain and digital competence. The academic contribution of the study lies in testing an integrated nursing work-system framework that simultaneously examines digital burden, digital capability, occupational strain, care-process reliability, and AI implementation readiness within the same analytic cohort, rather than evaluating any single domain in isolation.

Main contribution 1: A single-cohort analytic framework combining technostress, eHealth literacy, burnout, the safety climate, missed nursing care, and AI-DSS acceptance to test how these domains relate to one another.

Main contribution 2: Adjusted models estimating the independent associations of technostress and eHealth literacy with both care-process reliability and low AI-DSS acceptance.

Main contribution 3: Mediation and clustering analyses that move beyond simple bivariate associations to identify potentially explainable pathways and clinically interpretable high-risk workflow phenotypes.

Main contribution 4: Context-specific evidence from a Romanian tertiary-care hospital that informs implementation planning in settings undergoing digital transition rather than mature AI deployment.

## 2. Methods

### 2.1. Study Design and Setting

We conducted a cross-sectional survey of registered nurses working in a tertiary-care hospital in Timișoara, Romania, between January and March 2025. The study followed the STROBE reporting guidance for observational studies [15]. Eligible participants were registered nurses employed in inpatient units (critical care and general wards) for ≥6 months. Nurses on extended leave and agency/temporary staff were excluded. Unit managers disseminated the survey link and paper copies during staff meetings; participation was voluntary and anonymous. Of 267 nurses approached, 239 provided complete questionnaires (response rate 89.5%).

### 2.2. Ethical Considerations and Data Collection Procedure

The study protocol was approved by the Local Commission of Ethics of the “Pius Brinzeu” Clinical Emergency Hospital from Timișoara, Romania, and all procedures were conducted in accordance with the Declaration of Helsinki and applicable Good Clinical Practice principles. Participation was voluntary, anonymous, and uncompensated. After receiving study information, participants provided informed consent before completing the questionnaire. To reduce response inhibition and social desirability pressures, no direct supervisor had access to individual responses, questionnaires were completed privately, and only aggregated results were analyzed. Because all measures were collected in a single survey session, the study was designed to estimate associations rather than temporal or causal effects.

### 2.3. Measures and Instruments

Data were collected using a structured, self-administered questionnaire composed of validated instruments and study-specific items selected to capture digital strain, digital competency, occupational well-being, safety culture, care-process omissions, and openness to AI-supported clinical tools. All scales were administered in the same survey session, and higher scores were interpreted according to the conventions of each instrument. Internal consistency of all multi-item measures was evaluated using Cronbach’s alpha, with results indicating acceptable-to-excellent reliability across domains.

Technostress was assessed using a 15-item scale designed to measure the degree of strain experienced in relation to digital technologies used in daily clinical work. The instrument covered dimensions such as perceived overload, system complexity, frequent interruptions, pressure associated with digital documentation, and the intrusive spillover of technology into workflow organization. Items were scored on a 5-point Likert scale, with higher total scores indicating greater technostress. For analysis, technostress was examined both as a continuous score and as tertiles to facilitate subgroup comparisons and dose–response analyses.

eHealth literacy was measured using the 8-item eHealth Literacy Scale (eHEALS), a widely used instrument that evaluates perceived knowledge, comfort, and skills in identifying, understanding, appraising, and applying digital health information to solve health-related problems and support decision-making [1,2]. Each item was rated on a Likert-type response scale, and a total score was computed by summing item responses. Higher scores indicated greater perceived eHealth literacy and stronger readiness to engage with digital health resources.

Burnout was assessed with the Maslach Burnout Inventory–Human Services Survey (MBI-HSS), which captures three core dimensions of occupational burnout in healthcare workers: emotional exhaustion (EE), depersonalization (DP), and personal accomplishment (PA). Emotional exhaustion reflects feelings of being emotionally overextended and depleted by work demands; depersonalization refers to detached, impersonal, or cynical attitudes toward recipients of care; and personal accomplishment reflects perceived competence and effectiveness in one’s professional role. Subscale scores were calculated separately and treated as continuous variables in descriptive, correlational, and regression analyses.

Safety culture was evaluated using selected subscales from the Safety Attitudes Questionnaire (SAQ), specifically the teamwork climate and safety climate domains [10]. These subscales were chosen because they are particularly relevant to high-demand digital environments in which communication, coordination, and perceptions of organizational safety priorities may influence both care reliability and acceptance of new technologies. Higher SAQ scores indicated more favorable perceptions of teamwork and safety culture within the clinical unit.

Missed nursing care was measured using a 10-item missed care inventory assessing the frequency of required nursing tasks that were omitted or significantly delayed during the previous 7 days. The items covered essential care activities typically vulnerable to workload pressure, interruptions, or prioritization conflicts. A composite score was calculated to reflect the burden of care left undone, with higher values indicating more frequent omissions. This variable was used as a primary outcome because it represents a clinically meaningful process indicator of care quality and operational strain.

AI-DSS acceptance was assessed using a 6-item scale specifically focused on nurses’ perceptions of artificial intelligence-supported decision tools in clinical practice. The scale evaluated perceived usefulness, trust in AI-generated recommendations, and stated intention to use AI-supported outputs in patient care decisions if such tools were available in the local setting. Higher scores indicated greater attitudinal acceptance of AI-DSS and stronger implementation readiness. Accordingly, this construct reflects perceived readiness rather than observed usage behavior. Because the scale asked nurses whether they would use such tools if available, prior hands-on AI-DSS experience was not required; the construct therefore reflects perceived readiness and trust expectations rather than routine real-world exposure or use behavior. For multivariable modeling, both the continuous total score and a dichotomized indicator of low AI-DSS acceptance (defined as the lowest quartile) were used.

In addition to scale-based measures, the survey also collected participant characteristics, including age, sex, years of clinical experience, educational level, unit type, and shift-work status. A single-item measure assessed intention to leave the job within the next 12 months as a binary outcome. These variables were included to characterize the sample, compare critical care and general ward nurses, and adjust multivariable models for relevant occupational context.

### 2.4. Outcomes

The study evaluated two prespecified primary outcomes and several secondary outcomes designed to capture both care-process performance and readiness to engage with digital clinical support tools. The primary outcomes were: (i) missed nursing care count/score and (ii) AI-DSS acceptance. Missed nursing care was conceptualized as the frequency of required nursing activities that were omitted or substantially delayed during the previous 7 days because of insufficient time, workload pressure, competing priorities, or workflow disruption. The total missed care score was analyzed both as a continuous measure, reflecting the overall burden of care omissions, and descriptively across subgroups to characterize variation by unit context and digital strain profile. Higher values indicated more frequent missed or delayed essential nursing tasks and were interpreted as a marker of operational strain and reduced care-process reliability.

The second primary outcome, AI-DSS acceptance, reflected participants’ attitudinal openness to the use of artificial intelligence-supported clinical recommendations in routine nursing practice. This construct incorporated perceived usefulness, trust in AI-supported suggestions, and stated willingness to use such tools when available in the clinical environment. Higher total scores indicated more favorable implementation readiness. Because many institutions remain in early phases of AI deployment, AI-DSS acceptance was intentionally conceptualized as a perception-based readiness outcome rather than as documented use behavior. A secondary analytical approach classified low AI-DSS acceptance as membership in the lowest quartile of the acceptance scale for use in logistic regression models.

The secondary outcomes included the three burnout dimensions derived from the MBI-HSS—emotional exhaustion (EE), depersonalization (DP), and personal accomplishment (PA)—as continuous psychological outcomes relevant to occupational strain in digitally intensive work settings. In addition, intention to leave the job within the next 12 months was assessed as a binary workforce stability outcome (yes/no), given its potential relevance to both burnout and technology-related work burden.

To further clarify hypothesized pathways, missed nursing care and AI-DSS acceptance were treated as downstream outcomes influenced by technostress, eHealth literacy, burnout, and safety culture. Burnout and safety climate were additionally examined as intermediary outcomes in mediation analyses, while intention to leave was modeled as an exploratory consequence of cumulative strain. Together, these outcomes were selected to reflect a multilevel framework spanning individual well-being, care quality, workforce retention risk, and readiness for safe digital and AI integration in hospital nursing practice.

### 2.5. Statistical Analysis

Statistical analyses were conducted in R (v4.3). Approximately normally distributed continuous variables were summarized as mean ± SD, skewed continuous variables as median (IQR), and categorical variables as counts and percentages. Between-group comparisons used Student’s *t* tests for approximately normal continuous variables, Mann–Whitney U tests for skewed continuous variables, and χ^2^ tests for categorical variables. Pearson correlations quantified pairwise linear associations among the main study variables. Multivariable linear regression modeled missed nursing care, and multivariable logistic regression modeled low AI-DSS acceptance (lowest quartile). Predictors were selected a priori from the conceptual framework and prior literature to represent digital strain, digital competency, unit context, and safety climate while keeping models parsimonious relative to sample size. Mediation analyses (bootstrapped 5000 resamples) evaluated whether burnout EE and SAQ safety climate statistically accounted for the technostress–missed care association; given the cross-sectional design, these mediation results were interpreted as statistical decomposition of associations rather than causal pathways. K-means clustering was performed on standardized technostress, eHEALS, and burnout EE scores. We selected k = 3 as an exploratory and clinically interpretable solution representing lower-, intermediate-, and higher-strain phenotypes; however, formal internal validation metrics (e.g., silhouette width) were not available and the cluster solution should therefore be interpreted descriptively. ROC analysis evaluated discrimination for intention to leave using fitted probabilities from a model including technostress, emotional exhaustion, and missed nursing care. Because the ROC analysis was exploratory and intended only to describe in-sample discrimination within the present dataset, no train/test split or k-fold cross-validation was performed. The reported AUC should therefore be interpreted as optimistic and hypothesis-generating, not as validated predictive performance for deployment. All tests were two-sided with *p* < 0.05 considered statistically significant. To mitigate common method bias at the design stage, data collection was anonymous, no direct supervisor had access to individual responses, and conceptually distinct constructs were measured with separate validated instruments.

## 3. Results

Among 239 nurses (77.0% female; mean age 34.0 ± 8.0 years), 60 (25.1%) worked in critical care and 179 (74.9%) in general wards. Critical care nurses reported a higher median missed care count than ward nurses (3 vs. 2; *p* = 0.04) with otherwise similar demographics and safety attitude scores (Table 1). Table 1 summarizes the cohort and compares critical care with general wards. Groups were similar in age, sex, education, and safety attitude scores, but critical care nurses reported more missed nursing care (median 3 vs. 2; *p* = 0.04), consistent with higher task density in ICU workflows.

Table 2 reports mean scale scores and reliability. Internal consistency was acceptable to excellent across all instruments (Cronbach’s α 0.78–0.92), supporting use of composite scores in downstream modeling. The highest reliability was observed for burnout—emotional exhaustion (α = 0.92) and technostress (α = 0.91).

Table 3 demonstrates a graded relationship across technostress tertiles. Higher technostress aligned with lower eHealth literacy and AI-DSS acceptance, and with higher emotional exhaustion and missed nursing care (all *p*-trend < 0.001), suggesting a coherent high-strain profile.

Table 4 shows bivariate correlations. Technostress correlated positively with emotional exhaustion (r = 0.52) and missed care (r = 0.41), and negatively with AI-DSS acceptance (r = −0.30). eHealth literacy correlated positively with AI acceptance (r = 0.35) and negatively with technostress (r = −0.34). Safety climate correlated inversely with missed care (r = −0.37).

Table 5 presents adjusted predictors of missed nursing care. Technostress and emotional exhaustion were independently associated with higher missed care, while higher safety climate was associated with lower missed care. ICU status retained a smaller but significant association after adjustment, indicating contextual effects beyond individual scores.

Table 6 presents the multivariable logistic regression model for low AI-DSS acceptance (lowest quartile). Higher technostress and higher emotional exhaustion were associated with higher odds of low acceptance, while higher eHealth literacy and better teamwork were associated with lower odds. Discrimination was acceptable (C-statistic = 0.74).

Table 7 characterizes three workflow phenotypes. The low-strain/high-literacy cluster showed the best safety climate, lowest missed care, and highest AI acceptance. The high-strain/low-literacy cluster showed the opposite pattern and the highest intention to leave, supporting heterogeneity in digital workflow experiences beyond unit type. Because clusters were defined by technostress, eHealth literacy, and emotional exhaustion rather than demographic variables, the vulnerable phenotype should be identified using these measured work-system indicators rather than age or years of experience alone.

Table 8 summarizes mediation results. The technostress–missed care association was partially statistically accounted for by emotional exhaustion and, to a lesser extent, safety climate; combined indirect effects accounted for approximately 35% of the total association. These estimates should be interpreted as cross-sectional mediation and not as proof of causality.

Table 9 reports exploratory network edge estimates with bootstrap stability. The strongest positive edges linked technostress with emotional exhaustion and emotional exhaustion with missed care, while safety climate showed a moderate negative edge with missed care. eHealth literacy had the clearest positive edge with AI acceptance, reinforcing its potential protective role.

**Figure 1:**
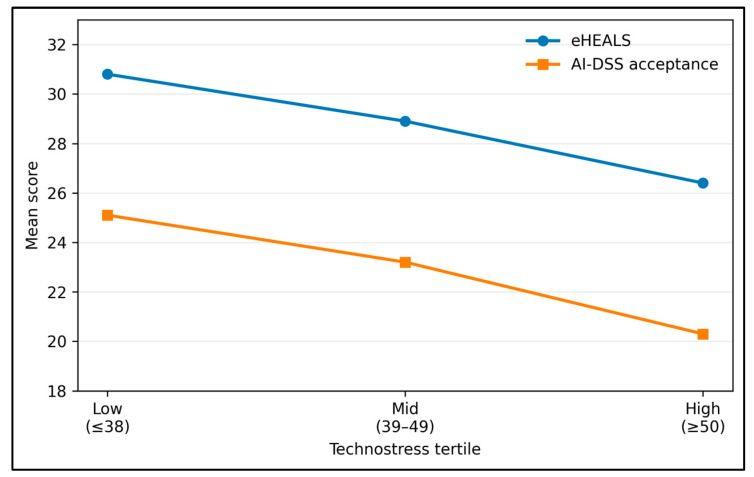
Mean eHealth literacy and AI-DSS acceptance scores across technostress tertiles.

Figure 1 provides a visual summary of the graded pattern already shown in Table 3. Mean eHEALS and mean AI-DSS acceptance scores both decline stepwise across the low-, mid-, and high-technostress tertiles. The figure therefore indicates that nurses in the highest technostress tertile reported both lower digital confidence and lower attitudinal readiness to use AI-supported decision tools. The figure is descriptive and should be interpreted as an intuitive visualization of group differences rather than as a causal model.

**Figure 2:**
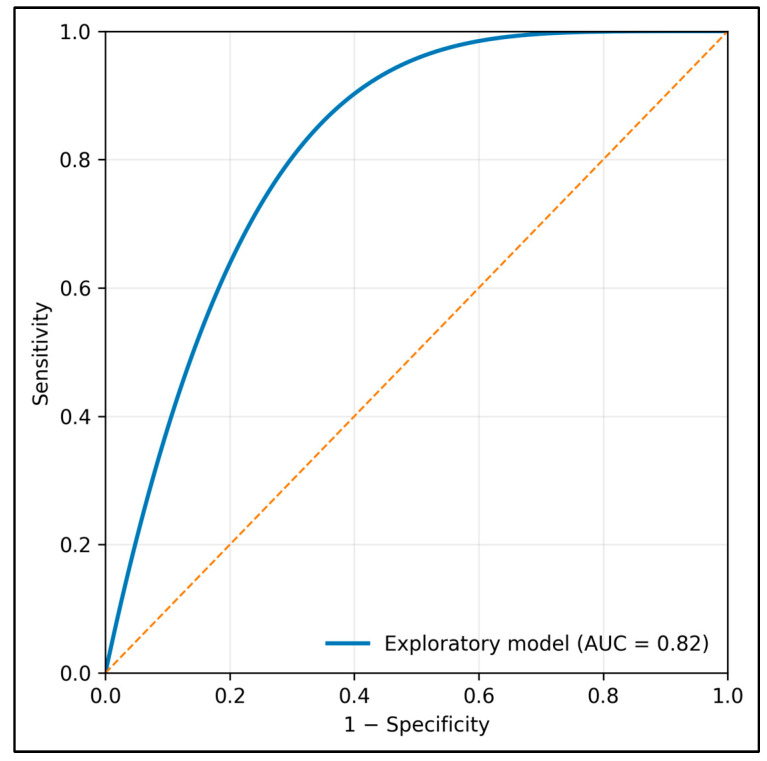
Exploratory receiver operating characteristic (ROC) curve for intention to leave the job.

Figure 2 displays the exploratory ROC curve corresponding to the reported model for intention to leave the job. The curve was derived from fitted probabilities generated by a model including technostress, emotional exhaustion, and missed nursing care and yielded an in-sample AUC of 0.82. Because no train/test split or k-fold cross-validation was performed, the figure should be interpreted as hypothesis-generating in-sample discrimination rather than as a validated screening instrument for workforce decisions.

## 4. Discussion

### 4.1. Analysis of Findings

Our findings indicate that technostress was consistently associated with both nurse well-being and frontline care processes. Higher technostress correlated with more emotional exhaustion and more missed nursing care, and it remained independently associated with missed care after adjustment for age and unit type. Because the design was cross-sectional, these findings should be interpreted as associations rather than directional effects. Even so, the consistency of the correlation, regression, and cluster patterns supports the interpretation that digital work strain co-occurs with care-process vulnerability in this cohort.

eHealth literacy emerged as a potentially protective factor. Higher eHEALS scores were associated with lower technostress and higher AI-DSS acceptance, and they remained associated with lower odds of low AI-DSS acceptance in the adjusted model. This aligns with emerging evidence that nurses’ digital competencies, self-efficacy, and readiness influence their willingness to engage with AI-enabled systems, particularly when adoption is still perception-based rather than embedded in routine workflows [21,23,24].

Our results are broadly consistent with the recent nursing literature linking technostress with burnout, but they extend that literature by connecting digital strain to missed nursing care and AI implementation readiness in the same sample. Wirth et al. and Kopuz et al. reported that technostress is closely related to burnout and that technology competence may attenuate some of its adverse effects [7,22]. In the present study, emotional exhaustion was not only correlated with technostress but also statistically accounted for part of the technostress–missed care association, suggesting that occupational depletion may be one mechanism through which digital burden coexists with care omissions.

The AI-related findings also merit cautious interpretation. A recent Taiwanese study found that human–machine trust and social influence were central to nurses’ behavioral intention to adopt AI technology, whereas perceived job stress was not independently significant [21]. Our findings differ in showing that higher technostress was associated with lower AI-DSS acceptance. This difference may reflect the construct we measured—generalized digital work strain rather than AI-specific job stress—as well as the fact that our outcome captured attitudinal readiness in a setting with limited routine AI exposure. Similarly, recent reviews emphasize that AI should augment rather than replace nursing judgment and that training, governance, and user-centered implementation are necessary for acceptance [18,24].

Importantly, the AI-DSS outcome in the present study captured attitudinal readiness rather than verified real-world use. This distinction matters because favorable perceptions can coexist with low routine uptake when usability, workflow integration, training, governance, or accountability barriers remain unresolved. The questionnaire did not require previous direct AI-DSS use and was explicitly framed around willingness to use such tools if available. Accordingly, our AI-related findings should be interpreted as signals of the implementation climate and readiness, not as direct evidence of effective adoption behavior, routine trust calibration through repeated use, or clinical impact.

The safety climate also mattered. A higher-SAQ safety climate was associated with less missed care and contributed to a smaller indirect pathway in the mediation model. This is clinically relevant because hospitals do not implement AI tools in a vacuum: the same teamwork norms, communication patterns, and safety priorities that affect care reliability are likely to shape how nurses interpret and trust algorithm-supported recommendations. The remaining approximately 65% of the technostress–missed care association not statistically accounted for by burnout and the safety climate may plausibly reflect other unmeasured influences, including staffing ratios, patient acuity, task interruptions, the physical unit layout, documentation-system usability, leadership support, and local implementation resources. The cluster analysis reinforces this point by showing that the most vulnerable phenotype was characterized not only by high technostress, but also by lower literacy, a worse safety climate, more missed care, and greater intention to leave.

From an implementation perspective, our results support a dual strategy for safer AI adoption in nursing: first, reduce unnecessary digital burden through usability-oriented redesign, streamlined documentation, and accessible technical support; second, strengthen digital and AI-related competencies through structured training and protected learning time. In a nursing context, “digital skills support” should include hands-on training in navigating digital workflows, interpreting AI-generated risk scores or alerts, recognizing when algorithmic outputs conflict with bedside assessment, documenting decisions appropriately, and escalating concerns when recommendations appear clinically implausible. Protected supervised practice time is likely to be as important as one-off technical instruction. The literature increasingly suggests that readiness for digitalization depends on organizational support as much as on individual capability [18,19,23,24]. In that sense, AI-DSS acceptance in this study should be viewed as a work-system outcome rather than a purely personal attitude.

The exploratory ROC analysis suggests that technostress, emotional exhaustion, and missed care together discriminate intention to leave reasonably well within this sample. However, the model was evaluated on the full dataset without a holdout sample or cross-validation. It should therefore be interpreted as hypothesis-generating, useful for identifying potentially relevant retention risk signals, but not yet suitable for operational workforce screening or deployment decisions.

Overall, these data indicate that digital transformation is not neutral. In this Romanian tertiary-care sample, higher technostress clustered with worse emotional exhaustion, more missed care, and lower AI-DSS acceptance, while higher eHealth literacy showed the opposite pattern. The findings are most plausibly transferable to hospitals undergoing similar phases of digital maturation, staffing pressure, and incremental AI implementation. Differences in scope-of-practice rules, staffing ratios, EHR maturity, training provision, and local AI governance may modify the magnitude or even the direction of some associations across settings. For that reason, the present results should be used as context-sensitive implementation evidence rather than assumed to generalize automatically across regions or health systems. More specifically, caution is warranted when extrapolating these findings to smaller community hospitals, highly resourced health systems, or institutions with mature AI deployment, because differences in nurse staffing, patient acuity, informatics infrastructure, training capacity, and governance may materially alter the observed associations. The present results should therefore be interpreted as context-sensitive implementation evidence rather than universally generalizable effect estimates [25,26,27,28,29,30,31,32,33,34].

### 4.2. Strengths and Limitations

This study has several strengths, including a high response rate, the use of established instruments with acceptable-to-excellent internal consistency, and an integrative framework spanning digital strain, the safety climate, care omissions, and AI implementation readiness. Nevertheless, several limitations should frame interpretation. First, the cross-sectional design precludes temporal or causal inference, so the regression and mediation analyses identify statistical associations rather than verified directional effects. Second, the study was conducted in a single Romanian tertiary hospital, which may limit transferability to health systems with different staffing models, digital maturity, AI availability, or implementation governance; this concern is especially relevant for smaller community hospitals and systems with markedly different digital infrastructure or staffing configurations. Third, all variables were collected by self-report during one survey session, creating risks of common method variance, recall bias, and socially desirable responding. Procedural safeguards included anonymous participation, the absence of supervisor access to individual responses, and the use of validated multi-item instruments, but these safeguards cannot eliminate shared-method inflation. Fourth, AI-DSS acceptance captured attitudinal readiness rather than actual usage behavior; objective usage logs, implementation exposure, and post-implementation performance metrics were not available. Fifth, residual confounding remains possible because potentially relevant factors such as the nurse-to-patient ratio, local digital-system usability, training exposure, and organizational support were not modeled directly. Sixth, the clustering and ROC analyses were exploratory. The k = 3 cluster solution was selected for interpretability but was not supported by formal internal validation metrics such as silhouette width, and the ROC model was evaluated in-sample without cross-validation or external validation, which may produce optimistic performance estimates. Although the cluster analysis identified a high-strain/low-literacy phenotype, the present dataset does not justify assigning that group a fixed demographic stereotype; the most defensible targeting approach is to identify nurses with concurrent high technostress, lower eHealth literacy, and higher emotional exhaustion and then to tailor support at the work-system level. Additional post hoc common-method diagnostics were not evaluated. Finally, although internal consistency was good, measurement validity in this specific implementation context should still be interpreted cautiously, especially for study-specific AI acceptance items. Future longitudinal, multicenter studies should combine survey data with objective workflow, staffing, usability, and adoption measures.

## 5. Conclusions

In this cross-sectional cohort of 239 hospital nurses, higher technostress was consistently associated with more emotional exhaustion, more missed nursing care, and lower attitudinal acceptance of AI-DSS, whereas higher eHealth literacy was associated with more favorable AI-DSS acceptance. In adjusted analyses, each 10-point increase in technostress was associated with a 0.28-point-higher missed care score (equivalent to 0.14 points per 5-point increase) and 38% higher odds of low AI-DSS acceptance, while each 5-point increase in eHEALS was associated with 29% lower odds of low acceptance. Cluster findings reinforced this pattern: the high-strain/low-literacy phenotype had the poorest safety climate, the most missed care, and the lowest acceptance. These results suggest that successful nursing implementation of AI-supported decision tools depends on the surrounding work system, not on the technology alone. Hospitals should therefore pair AI deployment with usability-focused redesign, protected training time, and structured digital skills support that includes workflow-based training, interpretation of AI outputs within clinical judgment, and supervised practice. Given the single-center, self-report, and partly exploratory nature of the analyses, these results should be interpreted as implementation-relevant associations requiring longitudinal and multicenter confirmation.

## Figures and Tables

**Table 1:** Participant characteristics by unit type (critical care vs. general wards).

Characteristic	Critical Care (*n* = 60)	General Wards (*n* = 179)	*p*-Value
Age, years (mean ± SD)	34.9 ± 8.3	33.7 ± 7.9	0.31
Female sex, *n* (%)	46 (76.7)	138 (77.1)	0.95
Years of clinical experience (mean ± SD)	9.4 ± 7.2	8.7 ± 6.8	0.48
Shift work (rotating), *n* (%)	54 (90.0)	151 (84.4)	0.29
Bachelor’s degree or higher, *n* (%)	50 (83.3)	142 (79.3)	0.49
SAQ safety climate (mean ± SD)	62.5 ± 11.4	65.2 ± 10.7	0.07
SAQ teamwork (mean ± SD)	67.1 ± 12.0	69.8 ± 11.2	0.10
Missed care count, median (IQR)	3 (2–5)	2 (1–4)	0.04

Abbreviations: IQR, interquartile range; SAQ, Safety Attitudes Questionnaire; SD, standard deviation.

**Table 2:** Scale scores and internal consistency (Cronbach’s α).

Domain/Scale	Items (*n*)	Mean ± SD	Cronbach’s α
Technostress (total)	15	44.2 ± 10.5	0.91
eHEALS	8	28.7 ± 5.6	0.89
Burnout—emotional exhaustion (EE)	9	23.5 ± 11.0	0.92
Burnout—depersonalization (DP)	5	7.9 ± 5.1	0.81
Burnout—personal accomplishment (PA)	8	34.1 ± 8.2	0.83
SAQ teamwork climate	6	68.9 ± 11.5	0.86
SAQ safety climate	7	64.5 ± 10.9	0.84
AI-DSS acceptance	6	22.9 ± 5.3	0.87
Missed nursing care	10	2.4 ± 1.7	0.78

Abbreviations: AI-DSS, artificial intelligence decision support system; eHEALS, eHealth Literacy Scale; EE, emotional exhaustion; DP, depersonalization; PA, personal accomplishment; SAQ, Safety Attitudes Questionnaire; SD, standard deviation.

**Table 3:** Key outcomes across technostress tertiles.

Technostress Tertile	*n* (%)	eHEALS (Mean ± SD)	Burnout EE (Mean ± SD)	Missed Care (Mean ± SD)	AI Acceptance (Mean ± SD)	*p*-Trend
Low (≤38)	78 (32.6)	30.8 ± 4.8	18.1 ± 9.3	1.6 ± 1.2	25.1 ± 4.6	<0.001
Mid (39–49)	83 (34.7)	28.9 ± 5.2	22.5 ± 10.1	2.3 ± 1.5	23.2 ± 5.1	
High (≥50)	78 (32.6)	26.4 ± 5.8	29.7 ± 10.8	3.2 ± 1.8	20.3 ± 5.4	

Abbreviations: eHEALS, eHealth Literacy Scale; EE, emotional exhaustion; SD, standard deviation.

**Table 4:** Correlations among technostress, eHealth literacy, burnout, safety climate, missed nursing care, and AI-DSS acceptance.

Variable	Technostress	eHEALS	Burnout EE	SAQ Safety Climate	Missed Care	AI Acceptance
Technostress	—	−0.34 ***	0.52 ***	−0.28 ***	0.41 ***	−0.30 ***
eHEALS	−0.34 ***	—	−0.22 **	0.19 **	−0.18 *	0.35 ***
Burnout EE	0.52 ***	−0.22 **	—	−0.31 ***	0.44 ***	−0.25 **
SAQ safety climate	−0.28 ***	0.19 **	−0.31 ***	—	−0.37 ***	0.21 **
Missed care	0.41 ***	−0.18 *	0.44 ***	−0.37 ***	—	−0.26 **
AI acceptance	−0.30 ***	0.35 ***	−0.25 **	0.21 **	−0.26 **	—

Abbreviations: AI-DSS, artificial intelligence decision support system; eHEALS, eHealth Literacy Scale; EE, emotional exhaustion; SAQ, Safety Attitudes Questionnaire. Significance: * *p* < 0.05; ** *p* < 0.01; *** *p* < 0.001.

**Table 5:** Multivariable linear regression predicting missed nursing care.

Predictor	β (SE)	95% CI	*p*-Value
Technostress (per 10 points)	0.28 (0.06)	0.16 to 0.40	<0.001
Burnout EE (per 10 points)	0.22 (0.05)	0.12 to 0.33	<0.001
SAQ safety climate (per 10 points)	−0.19 (0.07)	−0.33 to −0.05	0.009
ICU vs. ward	0.21 (0.10)	0.01 to 0.41	0.04
Age (per 10 years)	0.05 (0.04)	−0.03 to 0.13	0.21
Model adjusted R^2^	0.31		

Abbreviations: β, standardized coefficient; CI, confidence interval; ICU, intensive care unit; SAQ, Safety Attitudes Questionnaire.

**Table 6:** Multivariable logistic regression predicting low AI-DSS acceptance (lowest quartile).

Predictor	OR	95% CI	*p*-Value
Technostress (per 10 points)	1.38	1.16–1.64	0.001
eHEALS (per 5 points)	0.71	0.58–0.86	<0.001
Burnout EE (per 10 points)	1.22	1.03–1.44	0.02
SAQ teamwork (per 10 points)	0.84	0.71–0.99	0.04
ICU vs. ward	1.12	0.63–1.98	0.70
Model C-statistic	0.74		

Abbreviations: AI-DSS, artificial intelligence decision support system; CI, confidence interval; eHEALS, eHealth Literacy Scale; ICU, intensive care unit; OR, odds ratio; SAQ, Safety Attitudes Questionnaire.

**Table 7:** Workflow phenotypes identified by cluster analysis and associated outcomes.

Characteristic	Cluster 1 (*n* = 86)	Cluster 2 (*n* = 72)	Cluster 3 (*n* = 81)	η^2^	*p*-Value
eHEALS score (mean ± SD)	32.1 ± 4.2	27.8 ± 5.1	25.8 ± 5.4	0.24	<0.001
Technostress total (mean ± SD)	36.4 ± 6.9	44.7 ± 8.0	52.1 ± 7.4	0.31	<0.001
Burnout EE (mean ± SD)	17.2 ± 8.4	24.3 ± 10.2	29.8 ± 10.7	0.28	<0.001
SAQ safety climate (mean ± SD)	69.3 ± 9.8	64.1 ± 10.6	60.5 ± 11.1	0.09	<0.001
Missed care count (mean ± SD)	1.3 ± 1.0	2.4 ± 1.4	3.5 ± 1.8	0.34	<0.001
AI-DSS acceptance (mean ± SD)	26.0 ± 4.3	23.1 ± 4.9	19.7 ± 5.2	0.21	<0.001
Intention to leave, *n* (%)	9 (10.5)	15 (20.8)	26 (32.1)	0.08	0.002

Abbreviations: AI-DSS, artificial intelligence decision support system; EE, emotional exhaustion; SAQ, Safety Attitudes Questionnaire; SD, standard deviation.

**Table 8:** Mediation analysis of technostress and missed nursing care via burnout and safety climate.

Effect	Estimate (95% CI)	*p*-Value
Total effect (technostress → missed care)	0.42 (0.30–0.55)	<0.001
Direct effect	0.27 (0.14–0.41)	<0.001
Indirect via burnout EE	0.10 (0.06–0.16)	<0.001
Indirect via SAQ safety climate	0.05 (0.01–0.10)	0.02
Serial indirect (EE → safety climate)	0.02 (0.00–0.05)	0.06
Proportion mediated (combined)	35%	

Abbreviations: CI, confidence interval; EE, emotional exhaustion; SAQ, Safety Attitudes Questionnaire.

**Table 9:** Network model edge estimates and stability for central associations.

Network Component	Model Estimate	Bootstrap Mean	Bootstrap 95% CI	Stability (CS)
Technostress–Burnout EE	0.34	0.33	0.25 to 0.41	0.62
Burnout EE–Missed care	0.28	0.28	0.19 to 0.37	0.59
SAQ safety climate–Missed care	−0.22	−0.22	−0.31 to −0.13	0.55
eHEALS–AI acceptance	0.30	0.30	0.21 to 0.39	0.64
Technostress–AI acceptance	−0.18	−0.18	−0.27 to −0.09	0.57

Abbreviations: AI, artificial intelligence; CI, confidence interval; CS, correlation stability; EE, emotional exhaustion; SAQ, Safety Attitudes Questionnaire.

## Data Availability

The data presented in this study are available on request from the corresponding author. The data are not publicly available due to privacy or ethical restrictions.
